# Development of an oxide-dispersion-strengthened steel by introducing oxygen carrier compound into the melt aided by a general thermodynamic model

**DOI:** 10.1038/srep38621

**Published:** 2016-12-12

**Authors:** Mohammad Amin Moghadasi, Mahmoud Nili-Ahmadabadi, Farsad Forghani, Hyoung Seop Kim

**Affiliations:** 1 School of Metallurgy and Materials Engineering, University of Tehran, P.O. Box 11155-4563 Tehran, Iran; 2 Center of Excellence for High Performance Materials, University of Tehran, P.O. Box 14395-731, Tehran, Iran; 3 Department of Materials Science and Engineering (POSTECH), Pohang, 37673, South Korea

## Abstract

In general, melting process is not a common method for the production of oxide dispersion strengthened (ODS) alloys due to agglomeration and coarsening of oxide particles. However, vacuum casting process has recently been employed as a promising process to produce micro-scale oxide dispersed alloys. In this paper, we report the process and characterization of *in situ* formation and uniform dispersion of nano-scale Y-Ti oxide particles in Fe-10Ni-7Mn (wt.%) alloy. The processing route involves a solid-liquid reaction between the added TiO_2_ as an oxygen carrier and dissolved yttrium in liquid metal leading to an optimal microstructure with nano-sized dispersed oxide particles. The developed thermodynamic model shows the independence of the final phase constituents from experimental conditions such as melting temperature or vacuum system pressure which offers a general pathway for the manufacture of oxide dispersion strengthened materials.

Oxide dispersion strengthened (ODS) alloys, usually produced by a dispersion of nano-sized rare earth metal oxides such as yttrium oxides, are thriving structural materials for high temperature applications because they exhibit good mechanical properties such as tensile and creep strength at high temperatures^1^,^2^,^3^,^4^,^5^,^6^,^7^,^8^. Although powder metallurgy is the most frequent method for the manufacture of ODS alloys, in the present work, Fe-Ni-Mn alloy is selected as a tentative matrix to investigate a new ODS production procedure through vacuum casting route^9^,^10^,^11^.

The main difficulties of manufacturing oxide dispersion strengthened (ODS) alloys by casting processes are the control of oxide size in the molten metal and prevention of coarsening and agglomeration of oxide particles during melting and solidification^12^,^13^. The primary reason for oxide accumulation and coarsening during casting of iron based alloys is the high thermal stability of rare earth oxides such as Y_2_O_3_ at casting temperatures and thus high interface energy and poor wettability between these particles and molten Fe^14^. Specifically, yttrium oxide melting point is 2410 °C, and the high temperature contact angle of pure electrolytic iron drops in contact with yttrium oxide was approximately 90°^15^. This prevents the physical bonding between Y_2_O_3_ and the melt alloying elements. Nonetheless, vacuum casting technology has been suggested as an alternative method for fabrication of micro-scale ODS alloys^16^,^17^,^18^,^19^,^20^. Recently, the effectiveness of the introduction of micro-sized Y_2_O_3_ particles to the Fe-9Cr steel matrix through vacuum casting has been reported^20^. However, this steel has been characterized in heat treated condition, the size of the oxides particles is supposed to be almost similar to the as-cast samples. Actually, several investigations on Y-Ti oxide thermal stability demonstrate little coarsening during heat treatment at and below 1523 K^21^,^22^. As a case in point, experimental investigations conducted by Alinger^23^,^24^, show that negligible increase of Y-Ti nano-oxide size (from 2.64 nm to 2.76 nm) occurs at 1423 K after 3 hours. Therefore, it can be concluded that Y-Ti-O particle size is very dependent on melting and solidification through casting method while heat treatment in particular for a few hours, is not crucial and has not a substantial impact on the coarsening.

Recently, oxygen carrier concept has been developed in mechanically alloyed oxide dispersion strengthened (ODS) steels as a promising process to produce nanostructured ferritic alloys (NFAs)^25^,^26^,^27^,^28^,^29^. In this method, the addition of oxides which are less stable than Y_2_O_3_ provide an alternate way to form finely distributed and nanometer-sized Y-Ti-O complex oxides in α-Fe matrix as a result of internal oxidation^30^. A distinct advantage of *in situ* reaction procedure is the formation of micro-scale oxide dispersed alloys with a wide range of size and composition particles such as the Y-Ti-O complex oxides. Moreover, recent investigations demonstrate that the volume fraction and size distribution of oxide particles can be optimized by adjusting Ti and the extra oxygen content of the alloy^2^,^26^,^27^,^31^,^32^,^33^,^34^. Inspired by these achievements, we used TiO_2_ as a source of oxygen and dissolved Y in the liquid metal as a source of yttrium to produce nano-scale Y-Ti-O complex oxides through vacuum casting route. TiO_2_ was selected as an oxygen carrier due to its more wettability with molten metal compared to Y_2_O_3_ to control oxide distribution and prevent coarsening and agglomeration of oxide particles during the process.

Here, we elucidate the microstructural characteristics and second-phase particles in Fe-10Ni-7Mn (wt.%) alloy system as the consequence of the addition of yttrium followed by TiO_2_ oxygen carrier. The addition of yttrium changes the Fe-10Ni-7Mn microstructure by the formation of noticeable amount of lamellar precipitates and then the addition of TiO_2_ causes the formation of nano-sized Y-Ti-O and sub-micron Y_2_O_3_. These particles have been identified in details by means of electrolytic extraction of precipitates. Furthermore, a thermodynamic model is developed to predict the final phase constituents of systems which may be used to tailor the oxygen carrier technique for other alloy systems produced through vacuum casting.

## Thermodynamic model

We used a thermodynamic modeling to predict the final phase constituents of the alloy manufactured by oxygen carrier method. With regard to this fact, the formation of various Y and Ti oxide phases during solidification process was thermodynamically investigated. According to literature, three ternary oxide phases of Y_2_TiO_5_, Y_2_Ti_2_O_7_, and fluorite-type solid solution (FSS) exist in TiO_2_-Y_2_O_3_ system^35^,^36^. The FSS phase is a stoichiometric compound with the composition of 45 mol% TiO_2_ in the TiO_2_-Y_2_O_3_ system i.e. 9TiO_2_:11Y_2_O_3_ (with the Y_22_Ti_9_O_51_ formula)^37^. In the present model, the free energy of Y_2_O_3_, TiO_2_, FSS, Y_2_TiO_5_, Y_2_Ti_2_O_7_ oxides as well as FeNiMn-Y-Ti liquid phase have been computed concurrently to investigate the possible oxide formation during the solidification process. Table S3 demonstrates the parameters necessary for the CALPHAD formalism used to describe the free energies of the mentioned phases.

In order to determine the thermodynamic equilibrium phase separated state of the system, the total free energy of the system should be minimized by mediating the fraction of each phase. The total energy of system is determined by using the following equation


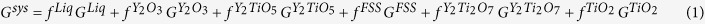


where f ^i^ is the mole fraction of phase i and G^i^ is the molar free energy of phase i. The superscript FSS and Liq refer to the fluorite solid solution phase and Fe-Ni-Mn-Y-Ti liquid phase, respectively. The free energy of liquid phase *G*^*Liq*^ is also calculated by


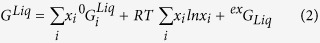


where R is the gas constant, x_i_ is the mole fraction of component i (i = Fe, Ni, Mn, Y and Ti) in the liquid phase, and 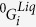 is the molar free energy of pure component i in the liquid state. The term 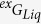 is the excess free energy of the mixing elements in the liquid phase, described by the Redlich-Kister polynomial^38^.


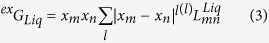


where m = Fe, Ni, Mn and n = Fe, Ni, Mn, Y, Ti. The parameters 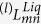 are the Redlich-Kister polynomial expansion coefficients listed in Table S3. Considering that Ti and Y in this work are generally dilute, Y–Y, Y–Ti, and Ti–Ti interactions in the liquid phase are neglected. With regard to the fact that neither Fe nor Ni and Mn are soluble in probable oxides of TiO_2_, Y_2_O_3_, Y_2_TiO_5_, Y_2_Ti_2_O_7_, and FSS, their value is assumed to be constant and only the value of Y and Ti in the liquid phase changes with regard to their consumption in the formation of oxides. In order to compare and investigate the accuracy of the thermodynamic calculations, the Gibbs free energy of the liquid phase has been recalculated using Thermo-Calc software in combination with TCFE7 database^39^. Finally, according to the aforementioned thermodynamic assumptions, the final equilibrium oxide phase separation in an Fe-Ni-Mn-Y-TiO_2_ alloy system can be determined by minimizing Eq.1 under the constraints of constant mole fraction of Fe, Ni, Mn, Y, and Ti.

## Results

### Microstructure

**Figure 1:**
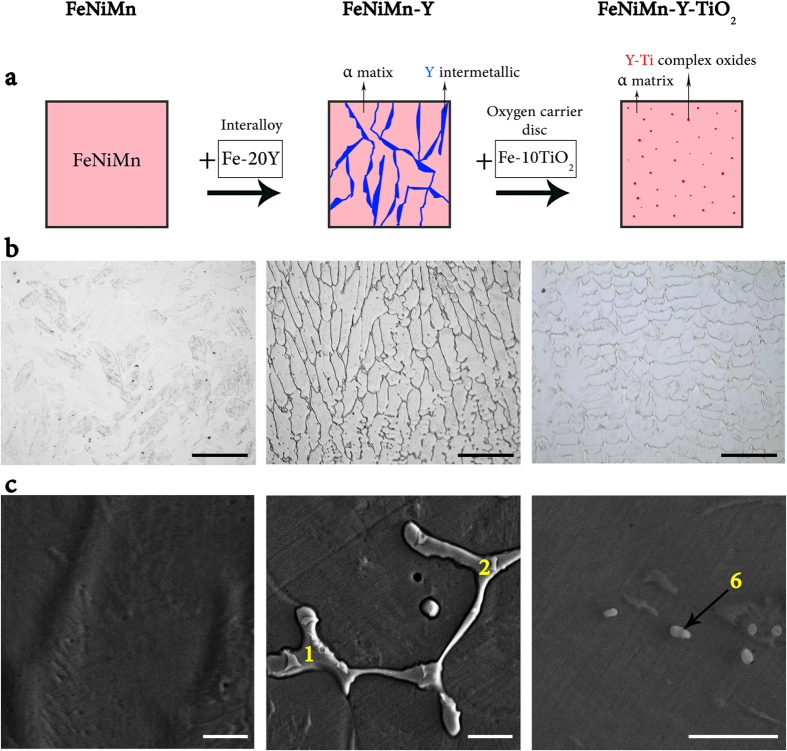
(**a**) Schematic illustration describing the simplicity and effectiveness of oxygen carrier method. (**b**) Optical (scale bars, 100 μm) and (**c**) SEM micrographs (scale bars, 5 μm) of the FeNiMn, FeNiMn-Y, and FeNiMn-Y-TiO_2_ as-cast specimens showing formation and disappearance of the lamellar precipitates as a result of yttrium and then oxygen carrier addition (EDS results of the points 1, 2 and 6 are shown in the Table S1).

Figure 1 shows a schematic representation and microstructural observation (optical microscope (OM) and scanning electron microscope (SEM) micrographs) of FeNiMn, FeNiMn-Y, and FeNiMn-Y-TiO_2_ specimens in as-cast condition. The figure indicates that while there is no evidence of precipitates in the microstructure of FeNiMn alloy, lamellar and continuous precipitates (about 10 vol.%) have been formed in FeNiMn-Y alloy as a consequence of yttrium addition. Furthermore, it can be seen that by the addition of TiO_2_ as an oxygen carrier, the continuous precipitates have disappeared again. Nevertheless, a few submicron precipitates can be recognized in the SEM figure of FeNiMn-Y-TiO_2_ alloy at higher magnification. It can also be expected that some small particles are not visible in the micrograph due to limited resolution of SEM.

The summary of the chemical compositions of precipitates numbered in Fig. 1c is listed in Table S1. Based on the Energy-dispersive spectroscopy (EDS) results of the precipitates in FeNiMn-Y specimen (numbers 1 to 5 in Table S1), the formed precipitates contain a significant percentage of iron, nickel, manganese, and yttrium, whereas the sub-micron precipitates in FeNiMn-Y-TiO_2_ specimen (number 6 in Table S1) contain a high percentage of yttrium and oxygen only. The slight differences in chemical compositions of the precipitates and also the small amount of iron in the results of the sub-micron particles can be attributed to the semi-quantitative nature of the EDS analysis as well as the inevitable effect of the matrix on the EDS analysis of small precipitates.

**Figure 2:**
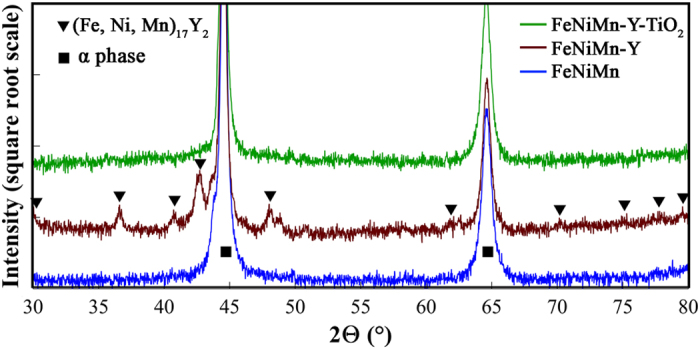
The XRD patterns of FeNiMn, FeNiMn-Y and FeNiMn-Y-TiO_2_ in the as-cast condition showing formation and disappearance of (Fe,Ni,Mn)_17_Y_2_ second phase particles due to the addition of Y and TiO_2_ oxygen carrier. (For better observation of the second phase weak peaks, the actual intensity of matrix peaks have been ignored).

The X-ray diffraction (XRD) patterns of FeNiMn, FeNiMn-Y, and FeNiMn-Y-TiO_2_ specimens are shown in Fig. 2. For better observation of the weak peaks, the intensity is presented in square root scale and also the actual height of α-Fe main peaks is ignored in this pattern. By comparing the XRD results of the three samples, it can be seen that by the addition of yttrium, the additional peaks of the second phase particle have appeared in FeNiMn-Y specimen. The observed peaks of these particles are identical to characteristic peaks of (Fe,Ni,Mn)_17_Y_2_ with hexagonal structure which has previously been reported in Fe-10Ni-7Mn-0.8Y alloy^40^. According to the results of EDS analysis (Table S1), the (Fe_0.7_Ni_0.22_Mn_0.08_)_17_Y_2_ stoichiometric compound can be proposed the precipitates of FeNiMn-Y alloy. Furthermore, the XRD pattern of FeNiMn-Y-TiO_2_ specimen shows that intermetallic peaks have vanished completely by adding TiO_2_; therefore, it can be concluded that addition of TiO_2_ oxygen carrier prevents the formation of intermetallic precipitates (Fe,Ni,Mn)_17_Y_2_ during solidification process. Although there is the possibility of the existence of some fine precipitates, with a higher ratio of yttrium and as a result with less volume fraction, these precipitates are not detectable within the XRD measurements accuracy.

### Characterization of Y-Ti-O particles

**Figure 3:**
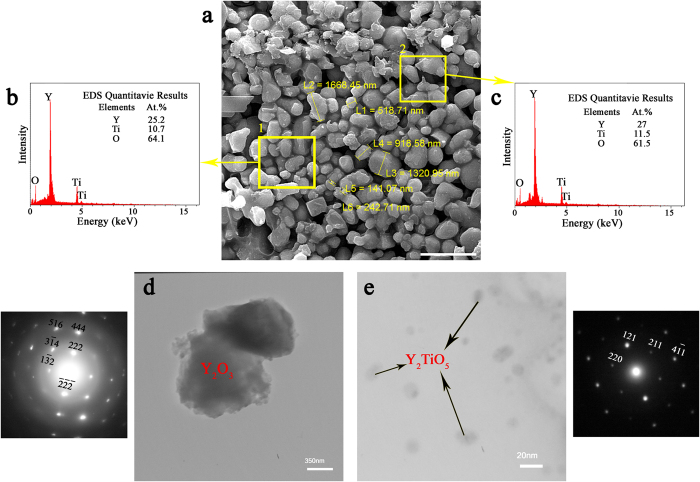
(**a**) FE-SEM image of extracted particles of the FeNiMn-1.6Y-1.8TiO_2_ as-cast specimen (scale bars, 3 μm). (**b**,**c**) Chemical composition of extracted precipitates measured by EDS spectrum corresponding to yellow rectangles in the FE-SEM image. (**d**) TEM image of the extracted particles with its SADP along [5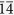] zone axis which confirms the formation of Y_2_O_3_ precipitates. (**e**) High magnification TEM image of extracted particles and its corresponding SADP along [1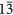] zone axis which shows the formation of nanometric Y_2_TiO_5_ precipitates.

To identify sub-micron particles in FeNiMn-Y-TiO_2_ alloy, the selective dissolution method was applied to dissolve metal elements, but to retain the oxide particles. The field emission scanning electron microscope (FE-SEM) image of the residue of the FeNiMn-Y-TiO_2_ specimen (Fig. 3a) shows the morphology and size distribution of sub-micron extracted particles. Figure 3b,c show the EDS analysis results of the extracted precipitates indicated by the yellow rectangles in Fig. 3a. Regarding this, yttrium, titanium, and oxygen are the main constituent elements of the extracted precipitates in FeNiMn-Y-TiO_2_ alloy. According to transmission electron microscopy (TEM) characterizations of the extracted particles, two different types of precipitates with average sizes of 11 ± 3 and 626 ± 188 nm have been formed in FeNiMn-Y-TiO_2_ alloy as a consequence of oxygen carrier addition (Fig. 3d,e). The size distribution of the extracted Y_2_O_3_ and Y_2_TiO_5_ particles are demonstrated in Fig. S1.

According to the EDS results of the extracted precipitates, there are four possible oxides containing yttrium and titanium elements, i.e. Y_2_O_3_, Y_2_TiO_5_, Y_2_Ti_2_O_7_, and TiO_2_^36^. Their structure characteristics are summarized in Table S2. Using the data of the atom positions, the crystal structures of probable oxides have been simulated and illustrated in Fig. S2. Based on drawn crystal structures, the diffraction patterns along their low index zone axes have been also calculated (Fig. S3) with the voltage and camera length of 150 keV and 1 m, respectively. We performed the diffraction simulation to index the selected area diffraction pattern (SADP) of different oxide particles observed in the TEM images. Figure 3d,e show the example of SADP indexing of two oxide particles. According to the results of electron diffraction study, it can be concluded that the coarse particles with an average size of about 626 ± 188 nm are Y_2_O_3_ and the fine particles with an average of size about 11 ± 3 nm are Y_2_TiO_5_ oxides (This is also confirmed by high resolution transmission electron microscopy (HRTEM) study of fine particles shown in Fig. S4).

**Figure 4:**
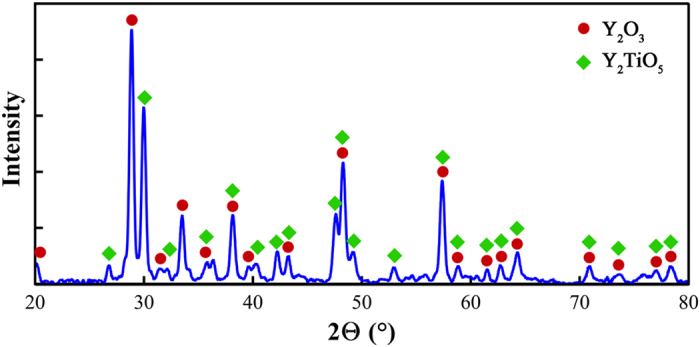
The XRD pattern of precipitates extracted from FeNiMn-Y-TiO_2_ specimen shown in SEM and TEM images (Fig. 3).

Cumulative results of TEM investigations and electron diffraction simulations reveal the formation of Y_2_O_3_ and Y_2_TiO_5_ oxides as a result of oxygen carrier addition. This is consistent with the X-ray diffraction results shown in Fig. 4, which clearly confirms the existence of Y_2_O_3_ and Y_2_TiO_5_ precipitates in the precipitates extracted from FeNiMn-Y-TiO_2_ alloy.

**Figure 5:**
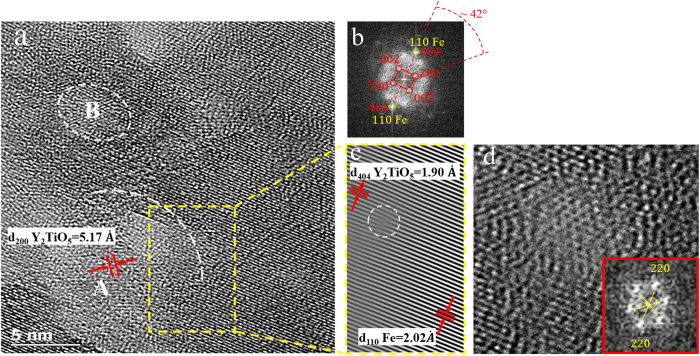
(**a**) HRTEM image of FeNiMn-1.6Y-1.8TiO_2_ specimen showing the existence of two Y_2_TiO_5_-type particles in α-Fe matrix. (**b**) FFT pattern of A region showing B = 010 of Y_2_TiO_5_ particle with orthorhombic crystal structure. (**c**) Inverse FFT of the yellow rectangle area revealing one misfit dislocation at the interface of oxide and α matrix. (**d**) HRTEM image of a Y_2_TiO_5_ particle with corresponding FFT pattern (B = 113).

The Y_2_TiO_5_ fine particles in the α-Fe matrix were also characterized using HRTEM method. In Fig. 5a two particles with less than 10 nm in size are recognizable in the α-Fe matrix. The fast Fourier transform (FFT) image of region A indicates that interplanar distances and angles are consistent with the crystallographic planes of Y_2_TiO_5_ structure (Fig. 5b). The overlapped spots of (404)_Oxide_ and 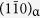 in Fig. 5b could suggest a cube-on-cube coherent relationship of the Y_2_TiO_5_ and the matrix. However, more detailed investigations are required to confirm this orientation relationship. Moreover, the inverse FFT image generated using 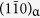 shown in Fig. 5c reveals a misfit dislocation (indicated by a white circle) at the coherent interface of Y_2_TiO_5_ and α matrix. This coherency is not too far-fetched since several studies demonstrate that complex Y-Ti oxides are found to be partially coherent with the ferritic matrix in ODS steels^5^,^41^,^42^,^43^. In this case, the partial lattice coherency between Y-Ti oxide and the steel matrix gives rise to low interface energy between the two disparate materials, oxide and metal. The low interface energy, along with very low solubility of O and Y in bcc Fe, can effectively reduce the coarsening rate of the oxide precipitates^44^.

## Discussion

The thermodynamic model described in Thermodynamic model section has been employed to make certain predictions and a rather general model for the FeNiMn-Y-TiO_2_ system behavior such as identities and stability of phases at equilibrium condition. We minimized the total free energy of the system under the constraints of experimental composition of the alloy, i.e. Fe ≈ 80.82, Ni ≈ 9.66, Mn ≈ 7.22, Y ≈ 1.02, and Ti ≈ 1.28 (at.%). In this regard, the Gibbs energy of Y_2_O_3_, TiO_2_, FSS, Y_2_TiO_5_, Y_2_Ti_2_O_7_ oxides as well as FeNiMn-Y-Ti liquid phase has been calculated to reach the equilibrium state of the system. In Fig. S5, the calculated Gibbs energy of the liquid phase is compared with the results of Thermo-Calc software, for instance. There is a good agreement between the results of current modeling and those of the software.

**Figure 6:**
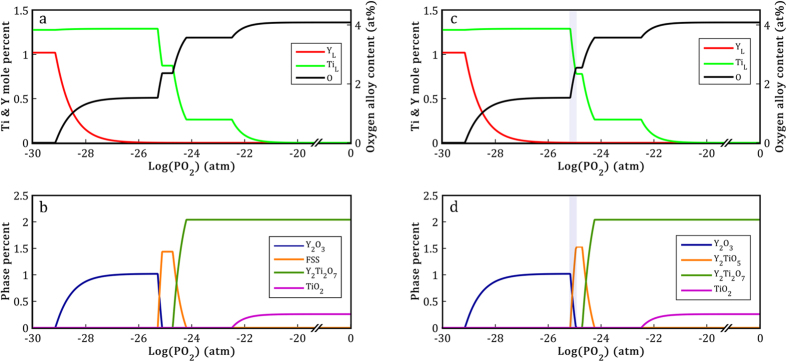
Equilibrium state prediction from the thermodynamic calculation at 1873 K in an FeNiMn-Y-TiO_2_ alloy. (**a**,**b**) Including FSS phase, (**c**,**d**) with regards to FSS decomposition into Y_2_O_3_ and Y_2_TiO_5_. (**a**,**c**) Dissolved Y and Ti in liquid phase on the primary axis and oxygen alloy content on the secondary axis as a function of the oxygen partial pressure. (**b**,**d**) Phase percentage of various stable Y-Ti oxides at different oxygen partial pressures.

The predicted stable phases of the FeNiMn-Y-TiO_2_ alloy at 1873 K against different oxygen partial pressures are shown in Fig. 6a,b. In Fig. 6a, the equilibrium solubility of Y and Ti in the liquid phase is demonstrated as a function of oxygen pressure. Figure 6b shows the phase percentage of the stable Y-Ti oxides in various oxygen pressures. Based on these thermodynamic calculations, TiO_2_, Y_2_Ti_2_O_7_, FSS (fluorite-type solid solution), and Y_2_O_3_ are the four probable stable Y-Ti oxides depending on the oxygen pressure of the system at 1873 K. All the mentioned oxide phases are stable up to the room temperature except for the FSS phase which decomposes into Y_2_O_3_ and Y_2_TiO_5_ by a eutectoid reaction at 1673 K^35^,^37^. Therefore, hereafter, the FSS phase has been ignored from thermodynamic modeling results in order to simplify the representation of the stable phases at room temperature.

Figure 6c,d show the thermodynamic equilibrium phase separated state of FeNiMn-Y-TiO_2_ system considering the above assumption. Generally, a reduction of oxygen pressure will result in a reduction of stable oxide compounds in the system. Simultaneously, there will be an increase in Y and Ti dissolution and there will also be more Y and Ti in the melted FeNiMn phase. In fact, these thermodynamic modeling results indicate that in the high oxygen pressure condition, TiO_2_ and Y_2_Ti_2_O_7_ are more stable oxides as expected from TiO_2_-Y_2_O_3_ pseudo-binary phase diagram under 1 atm^37^. By decreasing the oxygen pressure, TiO_2_ starts to decompose into O_2_ and dissolved Ti (TiO_2_ → Ti_L_ + O_2_); consequently, the concentration of Ti in the liquid phase increases slightly. After complete dissolution of TiO_2_ in the liquid phase, Y_2_Ti_2_O_7_ becomes the only stable oxide within limited range of oxygen pressure.

At much lower oxygen partial pressures, Y_2_Ti_2_O_7_ oxide also decomposes into Ti, Y_2_TiO_5_, and O_2_ (Y_2_Ti_2_O_7_ → Ti_L_ + Y_2_TiO_5_ + O_2_). After the complete consumption of Y_2_Ti_2_O_7_, Y_2_TiO_5_ is the only stable oxide within the small range of oxygen pressure. In the next step, as a result of further decrease in the oxygen pressure, Y_2_TiO_5_ starts to dissolve and concurrently Y_2_O_3_ starts to form (Y_2_TiO_5_ → Ti_L_ + Y_2_O_3_ + O_2_). By the end of this reaction, all the Ti content of the system is completely dissolved in the liquid phase and Y_2_O_3_ remains as the only stable oxide. By the continuation of the decline of oxygen pressure, Y_2_O_3_ can also decompose into O_2_ and dissolved Y (Y_2_O_3_ → 2Y_L_ + 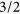
O_2_). Therefore, as shown in Fig. 6c, the amount of the dissolved Y in the liquid phase rises significantly. Finally, after complete decomposition of Y_2_O_3_, all the Y-Ti oxides disappear completely and the total amount of Y and Ti content is in the form of dissolved element in the FeNiMn liquid phase.

Due to the fact that solubility of oxygen for the FeNiMn-Y-Ti system in the liquid phase is negligible (less than 10 ppm for this work conditions according to the Sieverts’ law calculation), we have ignored it in the current modeling. Moreover, inasmuch as the Y and Ti elements exhibit higher affinity for oxygen compared with other elements of the system, it can be assumed that all the oxygen in the solidified alloy is in the form of Y-Ti oxides only. This assumption is in agreement with the previous results which have reported the formation of Y_2_O_3_ until complete consumption of the oxygen content in the yttrium-bearing alloys^40^,^45^. Therefore, the oxygen content of the solidified alloy could be calculated by considering the phase percentage of all Y-Ti oxides. This value has been also shown as a function of oxygen partial pressure by solid black lines in Fig. 6a,c.

**Figure 7:**
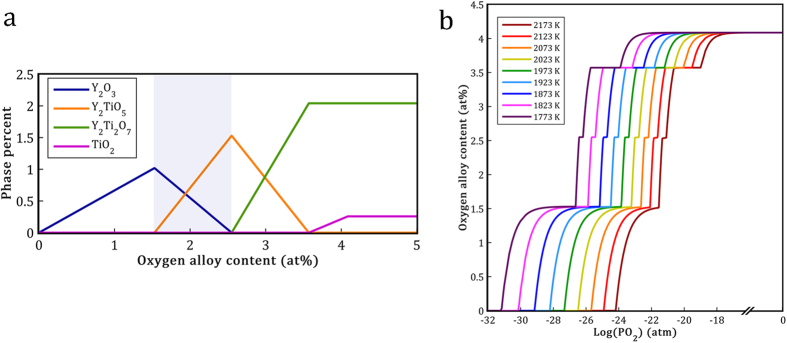
(**a**) Predicted equilibrium oxide phase percentage as a function of oxygen alloy content in the FeNiMn-Y-TiO_2_ system at 1873 K (the highlighted zone represents the Y_2_O_3_ and Y_2_TiO_5_ region which is consistent with the present work experimental results). (**b**) Oxygen alloy content present in Y-Ti oxides (oxygen alloy content) as a function of equilibrium oxygen pressure at different temperatures.

In Fig. 7a, the change in the oxides phase fraction of the FeNiMn-Y-TiO_2_ system is plotted as a function of the oxygen content. The highlighted regions in Figs 6c,d and 7a demonstrate the stability region of Y_2_O_3_ and Y_2_TiO_5_ oxides which were observed experimentally in this alloy. Based on the Fe-10Ni-7Mn-1.6Y-1.8TiO_2_ (wt.%) alloy composition, there is about 2.49 at.% oxygen in the alloy as a result of TiO_2_ oxygen carrier addition. Moreover, the former study performed on the FeNiMn-0.8Y alloy with similar processing conditions has revealed that only 0.116 at.% oxygen has entered the system from different sources such as raw material impurities or vacuum leakage during melting process^40^. Thus, all oxygen content in the current alloy should be around 2.61 at.% which is in reasonable agreement with the modeling results that indicate the maximum range of Y_2_O_3_ and Y_2_TiO_5_ region to be 2.63 at.% oxygen (Fig. 7a).

Figure 7b demonstrates oxygen alloy content curves of the FeNiMn-Y-TiO_2_ system at different temperatures. These curves show all the formation, stability, and decomposition steps of Y-Ti oxides. Therefore, this figure can be considered as a comprehensive representative of the system stability at different processing conditions. Surprisingly, it can be seen from the figure that changes in temperature and pressure of the system do not lead to a considerable change in the final phase constituents of the system. Instead, the equilibrium state of the system is mainly determined by the input value of oxygen in the form of TiO_2_ oxygen carrier. According to the figure, by changing the temperature, oxygen alloy content curves only shift slightly in the direction of horizontal axis. These displacements in the curves occurred within a very small range of the equilibrium oxygen pressure (between 10^−18^ to 10^−32^ atm). From the practical viewpoint, this negligible change of the equilibrium oxygen pressure is insensitive in this system. Thus, based on the thermodynamic results, it can be concluded that the formation of various complex Y-Ti oxides in the FeNiMn-Y-TiO_2_ system is independent of the experimental conditions such as melting temperature or vacuum furnace quality. This unique feature can propose the oxygen carrier method as a feasible method for manufacturing a new generation of ODS alloys.

**Figure 8:**
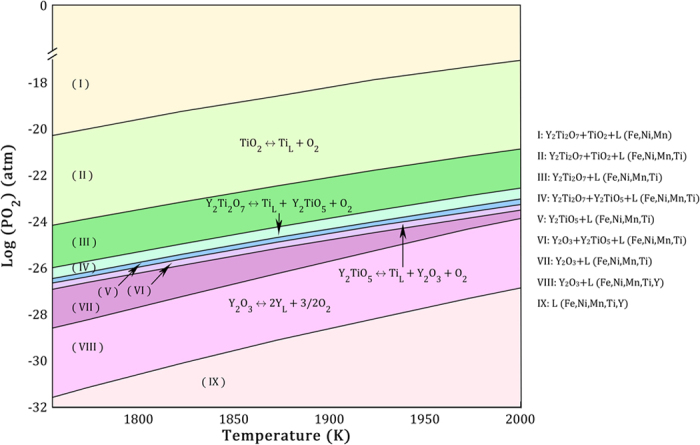
Equilibrium phases predicted by the thermodynamic model as a function of oxygen partial pressure 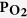 and temperature in the FeNiMn-Y-TiO_2_ alloy. Stable phases of each region are described in the right side of the figure. Reactions occurred in different regions are also indicated.

The calculated map of the equilibrium phases of the FeNiMn-Y-TiO_2_ system at different temperatures and oxygen partial pressures is shown in Fig. 8. The nine different regions can be recognized in the map. These regions can be divided into two different categories of stable and reaction regions. In stable regions, the amounts of the formed oxides as well as the liquid phase composition remain almost constant during oxygen pressure changes. But in reaction regions, the formation and decomposition of different Y-Ti oxides can occur simultaneously and as a result of this, the amount of dissolved Y or Ti can change in the liquid phase based on their consumption in the Y-Ti oxides. The map shows that with a decline in the oxygen pressure, five different stable regions containing various Y-Ti oxide phases, i.e. I: TiO_2_ + Y_2_Ti_2_O_7_; III: Y_2_Ti_2_O_7_; V: Y_2_TiO_5_; VII: Y_2_O_3_; and IX: without oxide appear sequentially. The four reaction regions occurring among these stable regions are also indicated in Fig. 8.

In the reaction region of VI, Y_2_TiO_5_ decomposes into Y_2_O_3_, O_2_, and dissolved Ti with a decline in the oxygen pressure. Thus, Y_2_TiO_5_, Y_2_O_3_, and the liquid phase which contains Fe, Ni, Mn, and Ti elements, are the stable phases in this region. The stable oxides of this region are consistent with the experimental results which show that Y_2_TiO_5_ and Y_2_O_3_ are the stable phases of the solidified FeNiMn-Y-TiO_2_ alloy. In the current system, regions IV, VI, and VIII are more important due to considerable variation of the oxygen alloy content occurred in these regions. It means that within a wide range of system oxygen content, as shown in Fig. 7a, the equilibrium state of the system is placed in these three regions. Thus, on the basis of current thermodynamic modeling, the stable phase constituents of each of these three regions can be easily achieved by controlling the oxygen content of the alloy as a result of oxygen carrier adjustment.

The importance of the described thermodynamic modeling as a numerical technique for the design of the new ODS alloys is apparent for the metallurgists. The developed model drawn upon available thermodynamic data can be easily employed to design new systems with different final phase constituents of Y-Ti oxides using oxygen carrier concept. Whether the oxygen carrier technique will have a beneficial effect on the high temperature properties of the alloys during creep deformation and how to adapt this concept to more complex commercial usable alloys will have to be shown in the future.

## Conclusion

In the present study, TiO_2_ oxygen carrier was utilized to produce ODS Fe-10Ni-7Mn alloy through vacuum casting process. The oxide precipitates were extracted electrolytically and their experimental characterization results were compared with those of crystal simulation. Moreover, a thermodynamic modeling framework was developed to study the thermodynamic aspect of the phase stability and oxide precipitation during the process. The principal conclusions derived from the experimental work and equilibrium thermodynamic model are as follows:

- Continuous and lamellar precipitates of (Fe,Ni,Mn)_17_Y_2_ intermetallic were formed as a result of yttrium addition to the FeNiMn alloy.

- Addition of TiO_2_ as an oxygen carrier to FeNiMn-Y prevented the formation of (Fe,Ni,Mn)_17_Y_2_ intermetallics and also led to the formation of the sub-micron and nano-sized Y-Ti oxides during solidification.

- As a result of oxygen carrier addition, the two different oxides of Y_2_O_3_ with an average size of 626 nm and Y_2_TiO_5_ with an average size of the 11 nm were characterized in FeNiMn-Y-TiO_2_ alloy. Moreover, the addition of TiO_2_ as an oxygen carrier succeeded in the size control and distribution of Y_2_O_3_ as well as the prevention of agglomeration and coarsening of oxides during the process.

- The thermodynamic modeling results demonstrate that there are nine different stability regions in the FeNiMn-Y-TiO_2_ system including four reaction regions and five stable regions.

- The precipitate volume fractions and phase identities predicted by the equilibrium thermodynamic model are in excellent agreement with experimental results. Therefore, the current thermodynamic model provides an adequate knowledge for understanding of formation mechanism of Y-Ti complex oxides and manufacturing new ODS alloys with controlled Y-Ti oxide constituents.

- Independence of thermodynamic equilibrium phase separated state from experimental conditions is an exclusive feature of this system which provides the possibility of development of the oxygen carrier concept to different alloy systems.

Our results suggest that by controlling oxygen content of an alloy using oxygen carrier, one can tailor oxide dispersion in the matrix and thereby control the properties of different alloy systems. Furthermore, the thermodynamic model proposed in this work can serve as a foundation for future modeling efforts to design intended ODS alloys with specified stable phases.

## Method

### Materials and manufacturing procedure

The three alloys investigated in this work are Fe-10Ni-7Mn, Fe-10Ni-7Mn-1.6Y, and Fe-10Ni-7Mn-1.6Y-1.8TiO_2_ (wt.%). These alloys were prepared by using the high purity raw materials including electrolytic Fe, electrolytic Mn, pure Ni shots, and pure Y pieces. Furthermore, Fe powder with an average particle size of 40 *μm* and TiO_2_ powder with an average particle size of 30 nm were used to produce Fe-10TiO_2_ (wt.%) oxygen carrier discs. The powders were mixed and milled in a stainless steel container with steel balls of 15 mm in diameter under argon atmosphere, a ball-to-powder weight ratio of 1:10 and at a mill speed of 250 rpm for a duration of 5 h. Ball milled powder was pressed with uniaxial pressure of 60Mpa and afterwards, the compressed discs were sintered at 1393 K for 5 h in a vacuum furnace. The FeNiMn alloy was prepared in a vacuum arc melting furnace under an argon gas atmosphere. The same technique was used to produce Fe-20Y (wt.%) interalloy by using electrolytic Fe and pure Y pieces. Afterwards, Fe-Ni-Mn Master alloy and the Y-rich interalloy were remelted in a vacuum arc remelting furnace to produce the FeNiMn-Y alloy. Finally, in order to prepare the FeNiMn-Y-TiO_2_ alloy, FeNiMn-Y ingot was remelted together with oxygen carrier discs in the vacuum arc remelting furnace under argon gas atmosphere.

### Characterization of microstructure

The microstructures of the samples were observed by means of OM (Zeiss Axioskop 2 MAT) and SEM (Tescan Vega), equipped with EDS (Oxford Instruments). The XRD analysis of bulk samples was performed using a Philips PW 1800 diffractometer (Cu Kα radiation of 40 kV and 50 mA) with small angular steps of 2θ = 0.02° and count time of 5 s per point to identify the precise intensity of weak peaks.

### Electrolytic extraction of particles

To eliminate any ambiguity about the chemical composition and crystal structure of sub-micron particles in the alloy, electrolytic phase extraction method was performed according to ASTM E963. In this process, the samples were anodically dissolved in an open cell with a platinum cathode and in a solution of 10% hydrochloric acid in methanol at a current density of 70 mA/cm^2^ in a glass beaker. Then, the residues were collected by centrifugal action at 4500 rpm for 10 min. After separation, the collected precipitates were dried in an oven at approximately 100 °C.

### Characterization and simulation of particles

The particles were analyzed by FE-SEM (Philips), XRD, and TEM (Philips CM30 and JEOL 2200FS). CrystalMaker and SingleCrystal software were employed to simulate the crystal structures and predict the electron diffraction patterns for possible structures. The simulated electron diffraction patterns were used to identify the experimental SAED and FFT patterns of the particles. TEM foils were prepared by mechanically polishing the samples to ~100 μm in thickness, followed by twin-jet electropolishing at 253 K in a solution of 10% perchloric acid and 90% acetic acid.

## Additional Information

**How to cite this article**: Moghadasi, M. A. *et al*. Development of an oxide-dispersion-strengthened steel by introducing oxygen carrier compound into the melt aided by a general thermodynamic model. *Sci. Rep.* **6**, 38621; doi: 10.1038/srep38621 (2016).

**Publisher's note:** Springer Nature remains neutral with regard to jurisdictional claims in published maps and institutional affiliations.

## Supplementary Material

Supplementary Information
