# Supplementary material for: Development of an oxide-dispersion-strengthened steel by introducing oxygen carrier compound into the melt aided by a general thermodynamic model
Source: Sci Rep. 2016 Dec 12;6:38621. doi: 10.1038/srep38621 (PMC5150259; doi:10.1038/srep38621)
Supplement: Supplementary Information [file srep38621-s1.pdf]

SUPPLEMENTARY INFORMATION:

Development of an oxide-dispersion-strengthened steel by introducing oxygen carrier compound into the melt aided by a general thermodynamic model

Mohammad Amin Moghadasi[1], Mahmoud Nili-Ahmadabadi[1,2,*], Farsad Forghani[1] & Hyoung Seop Kim[3]

[1]School of Metallurgy and Materials Engineering, University of Tehran, P.O. Box 11155-4563 Tehran, Iran.

[2]Center of Excellence for High Performance Materials, University of Tehran, P.O. Box 14395-731, Tehran, Iran.

[3]Department of Materials Science and Engineering (POSTECH), Pohang, 37673, South Korea.

*nili@ut.ac.ir

Participate Numbers	Elements (at. %)					
	**Fe**	**Ni**	**Mn**	**Y**	**Ti**	**O**
1	61.7	19.4	8.2	10.7	-	-
2	63.4	20.3	6.7	9.6	-	-
3*	63.2	18.9	7.6	10.3	-	-
4*	62.4	19 .1	8.1	10.4	-	-
5*	60.9	21.2	7	10.9	-	-
6	3.4	<0.1	<0.1	47.4	<0.1	49.2

Table S1. Chemical composition of precipitates shown in Fig. 1 measured by SEM-EDX. (*These analyses are not depicted in the figures.)

**Oxide**	**structure**	**Space group and no.**	**Lattice constant (Å)**	**Ref.**
Y_2O_3	Cubic	$Ia\bar{3}$, no. 206 (T_h^7)	a = 10.604	[1]
Y_2TiO_5	Orthorhombic	Pnma, no. 62 (T_{2h}^{16})	a = 10.35; b = 3.7; c = 11.25	[2]
$Y_2Ti_2O_7$	Cubic	$Fd\bar{3}m$, no. 227 (O_h^7)	a = 10.09397	[3]
TiO_2	Tetragonal	$P4_2$/mnm, no. 136 (D_{4h}^{14})	a = 4.58878; c = 2.95756	[4]

Table S2. Structure of possible oxides containing Y, O, and Ti in FeNiMN-Y-TiO_2 alloy.

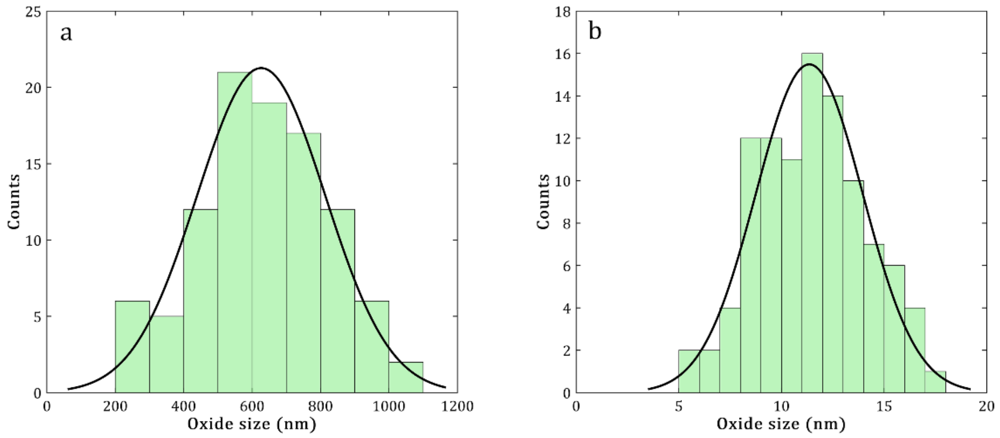

Figure S1. The size distribution of the extracted particles for a) Y_2O_3 with an average size of about 626 ±188 nm and b) Y_2TiO_5 with an average size of about 11 ±3 nm (One can note that there is still the possibility of the existence of finer Y_2TiO_5 particles in the alloy which were not collected during extraction method). The normal distribution curves are superimposed (the particle size is defined as $D = (4A/\pi)^{0.5}$, where A is the area of a particle. For each oxide, the area of 100 particles were measured by image analyzer software).

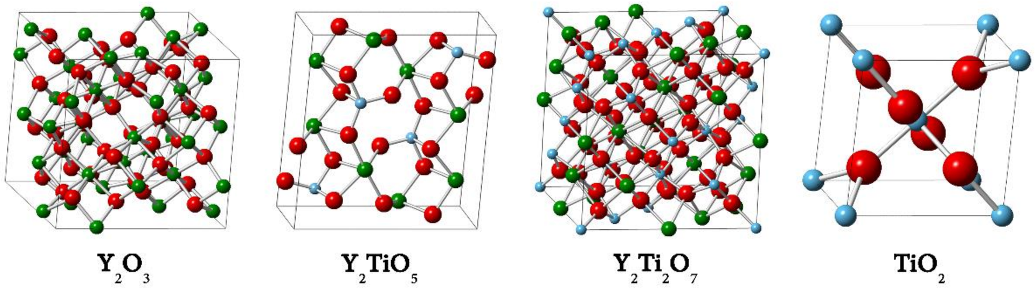

Figure S2. Simulated crystal structure of Y_2O_3, Y_2TiO_5, $Y_2Ti_2O_7$, and TiO_2 based on atom position data is shown in Table 4. Y, Ti, and O atoms are represented in green, blue and red, respectively.

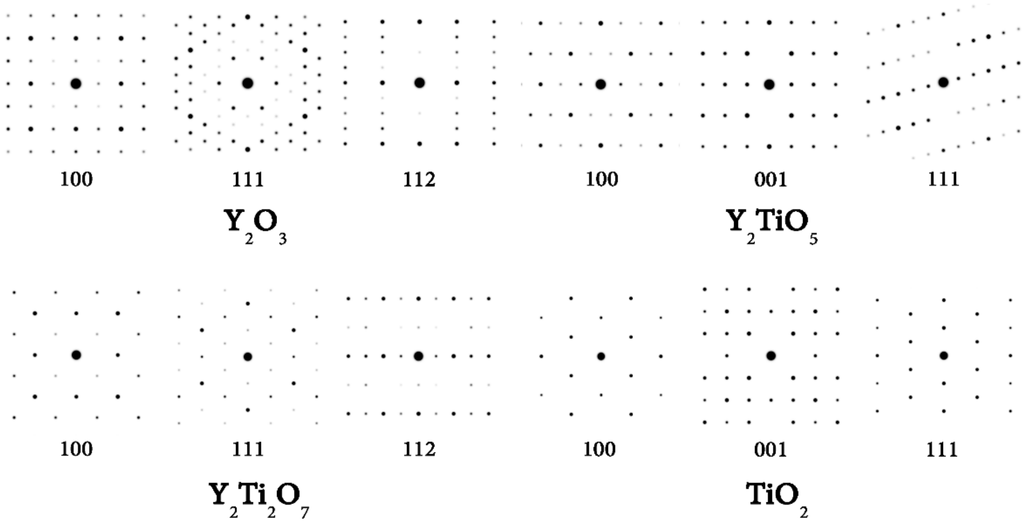

Figure S3. Calculated diffraction patterns of Y_2O_3, Y_2TiO_5, $Y_2Ti_2O_7$, and TiO_2 oxides low indexation. The voltage and camera length have been assumed to be 150 keV and 1 m, respectively.

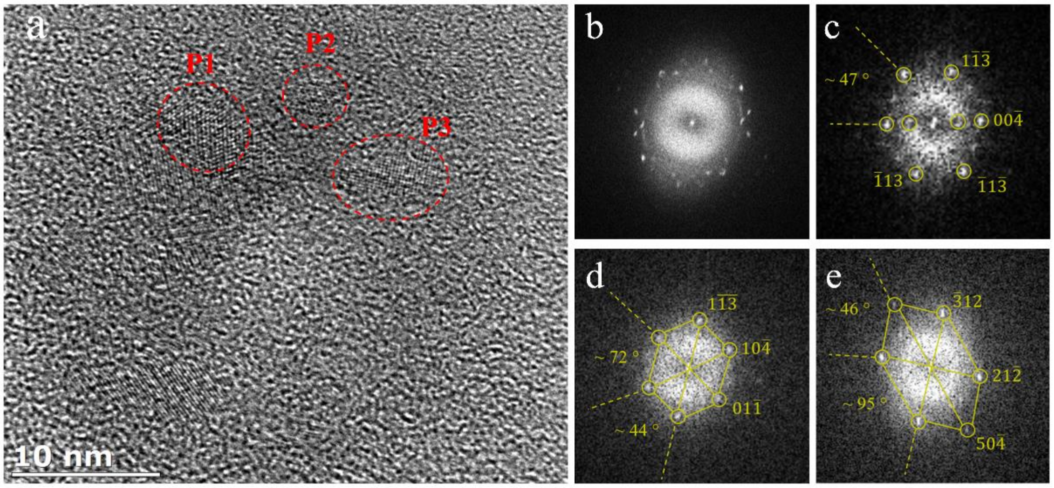

Figure S4. a) HRTEM image of particles extracted from FeNiMn-1.6Y-1.8TiO_2 specimen (some fine crystalline particles on the carbon support film are observable in the image). b) FFT of the image showing the mixed diffraction patterns of several particles. FFT patterns of particles c) P1, d) P2, and e) P3 show the B=110, B=410, and B=425 of the Y_2TiO_5 structure, respectively.

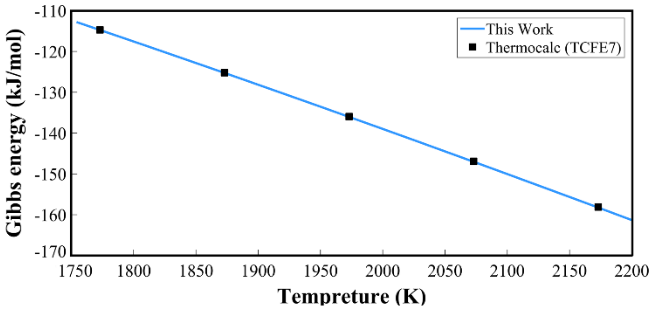

Figure S5. Calculated Gibbs energy of Fe-10Ni-7Mn-1Ti (at.%) liquid phase at different temperatures showing excellent agreement with Thermo-Calc (TCFE7) results.

Term	Expression	Temp. Rang	Ref.
${}^{0}G_{Fe}^{liq}$	$= 13265.87 + 117.57557\,T - 23.5143\,T\ln(T) - 0.00439752\,T^2 - 5.89269 \times 10^{-8}T^3$ $+77358.5\,T^{-1} - 3.6751551 \times 10^{-21}T^7$	298-1811K	5
	$= 10.838.83 + 291.302\,T - 46.0\,T\ln(T)$	1811-6000K	
${}^{0}G_{Ni}^{liq}$	$= 11235.527 + 108.457\,T - 22.096\,T\ln(T) - 4.8407 \times 10^{-3}T^2 - 3.82318 \times 10^{-21}T^7$	298-1728K	5
	$= -9549.775 + 268.598\,T - 43.10\,T\ln(T)$	1728-3000K	
${}^{0}G_{Mn}^{liq}$	$= 9744.63 + 117.4382\,T - 23.4582\,T\ln(T) - 7.3476 \times 10^{-3}\,T^2 + 69827.1\,T^1$ $-4.4192927 \times 10^{-21}\,T^7$	298-1519K	5
	$= -9993.9 + 299.036\,T - 48.0\,T\ln(T)$	1519-2000K	

${}^{0}G_{Y}^{liq}$	$= 3934.121 + 59.921688\,T - 14.8146562\,T\ln(T) - 15.623487\times10^{-3}\,T^2 + 1.442946\times10^{-6}\,T^3 - 140695\,T^{-1}$	298-1799K	5
	$= -13337.609 + 258.004539\,T - 43.0952\,T\ln(T)$	1799-3700K	
${}^{0}G_{Ti}^{liq}$	$= 4134.494 + 126.63427\,T - 23.9933\,T\ln(T) - 4.777975\times10^{-3}\,T^2 + 0.106716\times10^{-6}\,T^3 + 72636\,T^{-1}$	298-900K	5
	$= 4382.601 + 126.00713\,T - 23.9887\,T\ln(T) - 4.2033\times10^{-3}T^2 - 0.090876\times10^{-6}T^3 + 42680\,T^{-1}$	900-1155K	
	$= 13103.253 + 59.9956\,T - 14.9466\,T\ln(T) - 8.1465\times10^{-3}T^2 - 0.202715\times10^{-6}T^3 - 1477660\,T^{-1}$	1155-1300K	
	$= 369519.198 - 2554.0225\,T + 342.059267\,T\ln(T) - 163.409355\times10^{-3}T^2 + 12.457117\times10^{-6}T^3 - 67034516\,T^{-1}$	1300-1941K	
	$= -19887.066 + 298.7367\,T - 46.29\,T\ln(T)$	1941-4000K	
$G^{Y_2O_3}$	$= -1976462 + 731.6512\,T - 121.881\,T\ln T - 0.00506\,T^2 + 1090000 - 13000000\,T^{-2} - (3/2)\,RTlnP_{O_2}$		6
G^{TiO_2}	$= -976986.6 + 484.74037\,T - 77.76175\,T\ln T + 1683920\,T^{-1} - 67156800T^{-2} - RTlnP_{O_2}$		7
$G^{Y_2Ti_2O_7}$	$= G^{Y_2O_3} + 2G^{TiO_2} - 65569.9 - 37.19T$		8
$G^{Y_2TiO_5-}$	$= G^{Y_2O_3} + G^{TiO_2} - 27167.93 - 24.3.3T$	298-1603K	8
$G^{Y_2TiO_5-}$	$= G^{Y_2O_3} + G^{TiO_2} - 31976.9 - 21.3T$	T>1603K	8
G^{FSS}	$22G^{Y_2O_3} + 9G^{TiO_2} - 365.6T$		8
Redlich-Kisrer coefficients	${}^{(0)}L_{FeNi}^{Liq} = -18782 + 3.7011\,T$<br>${}^{(1)}L_{FeNi}^{Liq} = 12308.6 - 2.75998\,T$<br>${}^{(2)}L_{FeNi}^{Liq} = 4457 - 4.1536\,T$		9
	${}^{(0)}L_{NiMn}^{Liq} = -18782 + 3.7011\,T$<br>${}^{(1)}L_{NiMn}^{Liq} = 11665.5 - 7.6061\,T$		10
	${}^{(0)}L_{FeMn}^{Liq} = -2928.5 + 0.8779\,T$<br>${}^{(1)}L_{FeMn}^{Liq} = 849 + 0.3832\,T$		11
	${}^{(0)}L_{FeY}^{Liq} = -36095.134 - 3.602385\,T + 0.0016606922\,T^2$<br>${}^{(1)}L_{FeY}^{Liq} = -5490.9858 - 16.447786\,T$<br>${}^{(2)}L_{FeY}^{Liq} = 26444226 - 23.30126\,T$		12
	${}^{(0)}L_{NiY}^{Liq} = -155496 + 38.932\,T$<br>${}^{(1)}L_{NiY}^{Liq} = -52904 + 6.785\,T$		13
	${}^{(0)}L_{MnY}^{Liq} = -8613.87 + 0.71782\,T$<br>${}^{(1)}L_{MnY}^{Liq} = -2877.73 + 0.23981\,T$		14
	${}^{(0)}L_{FeTi}^{Liq} = -71347 + 8.25\,T$<br>${}^{(1)}L_{FeTi}^{Liq} = 7434 - 4.5\,T$<br>${}^{(2)}L_{FeTi}^{Liq} = 12155 + 0.25\,T$		15
	${}^{(0)}L_{NiTi}^{Liq} = -152500 + 43\,T$<br>${}^{(1)}L_{NiTi}^{Liq} = -52000 + 5.5\,T$<br>${}^{(2)}L_{NiTi}^{Liq} = -22500 + 4.125\,T$		16
	${}^{(0)}L_{MnTi}^{Liq} = -34000 + 21.5\,T$<br>${}^{(1)}L_{MnTi}^{Liq} = 1400$		17

Table S3. CALPHAD parameters used in the thermodynamic model (j/mol).

References

1. Ferreira, F. F. *et al.* X-ray powder diffraction beamline at D10B of LNLS: Application to the Ba2FeReO6 double perovskite. *J. Synchrotron Radiat.* **13,** 46–53 (2006).

2. Bevan, D. J. M. & Summerlivve, E. *Handbook on the physics and chemistry of rare earths*. **255,** (1979).

3. Chtoun, E., Hanebali, L., Garnier, P. & Kiat, J. M. X-rays and neutrons rietveld analysis of the solid solutions (1-x)A2Ti2O7-xMgTiO3 (A = Y or Eu). *Eur. J. solid state Inorg. Chem.* **34,** 553–561

4. Dorolti, E. *et al.* Half-metallic ferromagnetism and large negative magnetoresistance in the new lacunar spinel GaTi3VS8. *J. Am. Chem. Soc.* **132,** 5704–5710 (2010).

5. Dinsdale, A. T. SGTE data for pure elements. *Calphad* **15,** 317–425 (1991).

6. Djurovic, D., Zinkevich, M. & Aldinger, F. Thermodynamic modeling of the yttrium-oxygen system. **31,** 560–566 (2007).

7. Cancarevic, M., Zinkevich, M. & Aldinger, F. Thermodynamic description of the Ti-O system using the associate model for the liquid phase. *Calphad Comput. Coupling Phase Diagrams Thermochem.* **31,** 330–342 (2007).

8. Gong, W. *et al.* Phase equilibria of the TiO_2 - Y_2O_3 system. *Calphad* **33,** 624–627 (2009).

9. De Keyzer, J., Cacciamani, G., Dupin, N. & Wollants, P. Thermodynamic modeling and optimization of the Fe–Ni–Ti system. *Calphad-Computer Coupling Phase Diagrams Thermochem.* **33,** 109–123 (2009).

10. Guo, C. & Du, Z. Thermodynamic optimization of the Mn-Ni system. *Intermetallics* **13,** 525–534 (2005).

11. Witusiewicz, V. T., Sommer, F. & Mittemeijer, E. J. Reevaluation of the Fe-Mn phase diagram. *J. Phase Equilibria Diffus.* **25,** 346–354 (2004).

12. Kardellass, S. *et al.* A thermodynamic assessment of the iron-yttrium system. *J. Alloys Compd.* **583,** 598–606 (2014).

13. Du, Z. & Zhang, W. Thermodynamic assessment of the Ni-Y system. *J. Alloys Compd.* **245,** 164–167 (1996).

14. Gröbner, J., Pisch, A. & Schmid-Fetzer, R. Thermodynamic optimization of the systems Mn-Gd and Mn-Y using new experimental results. *J. Alloys Compd.* **317-318,** 433–437 (2001).

15. Dumitrescu, L. F. S., Hillert, M. & Sounders, N. Comparison of Fe-Ti assessments. *J. Phase Equilibria* **19,** 441–448 (1998).

16. Dupin, N. Thermodynamic study of multicomponent nickel-based systems. (1992).

17. Ansara, I., Dinsdale, a. T. & Rand, M. H. Thermochemical Database for Light Metal Alloys. (1998).
